# Early activation of Toll-like receptor-3 reduces the pathological progression of Alzheimer’s disease in APP/PS1 mouse

**DOI:** 10.1186/s13195-023-01186-w

**Published:** 2023-02-16

**Authors:** Shang Wang, Taiyang Zhu, Wanyan Ni, Chao Zhou, Hui Zhou, Li Lin, Yuting Hu, Xiaoyu Sun, Jingjing Han, Yan Zhou, Guoliang Jin, Jie Zu, Hongjuan Shi, Xingxing Yang, Zuohui Zhang, Fang Hua

**Affiliations:** 1 Institute of Neurological Diseases, Xuzhou Medical University, Xuzhou, China; 2 Department of Human Anatomy, Kangda College of Nanjing Medical University, Lianyungang, China; 3 Department of Neurology, Affiliated Hospital of Xuzhou Medical University, Xuzhou, China; 4 Department of Rehabilitation Medicine, Affiliated Hospital of Xuzhou Medical University, Xuzhou, China; 5 Department of Rehabilitation Medicine, The Second Affiliated Hospital of Nanjing Medical University, Nanjing, China; 6 Department of Interdisciplinary Health Science, College of Allied Health Science, Augusta University, Augusta, 30912 USA

**Keywords:** Alzheimer’s disease, TLR3, Amyloid β protein, Neuroinflammation

## Abstract

**Background:**

Toll-like receptor 3 (TLR3) plays an important role in the immune/inflammatory response in the nervous system and is a main pathological feature of Alzheimer’s disease (AD). This study investigates the role of early activation of TLR3 in the pathophysiological process of AD.

**Methods:**

In the experiment, the agonist of TLR3, Poly(I:C), was intraperitoneally injected into the APP/PS1 mouse model of AD and wild-type control mice starting from the age of 4 to 9 months. At the age of 14 months, behavioral tests were conducted. Western blot and immunohistochemistry staining were used to evaluate the level of amyloid β-protein (Aβ), the activation of inflammatory cells, and neuron loss. In addition, the levels of inflammatory cytokines were measured using a quantitative polymerase chain reaction.

**Results:**

The results demonstrated that the early activation of TLR3 attenuated neuronal loss and neurobehavioral dysfunction. Moreover, the early activation of TLR3 reduced Aβ deposition, inhibited the activation of microglia and astrocytes, and decreased the transcription of pro-inflammatory factors in the hippocampus.

**Conclusions:**

The results indicated that the activation of TLR3 by Poly (I:C) in the early stage of development of AD in a mouse model attenuated neuron loss and improved neurobehavioral functions. The underlying mechanisms could be attributed to its role in Aβ clearance, the inhibition of glial cells, and the regulation of neuroinflammation in the hippocampus.

## Background

Since the German psychiatrist Alois Alzheimer first reported it in 1907, Alzheimer’s disease (AD) has become a global public health concern [1]. Currently, about 40 million people worldwide suffer from Alzheimer’s disease. As the population ages, this number is expected to increase to 80 million by 2050 [2, 3]. However, there are still no effective methods for preventing and treating this disease. Moreover, its pathological mechanism remains incompletely understood.

Clinically, AD is characterized by progressive learning and memory deficits, as well as possible hallucinations and seizures, which ultimately impede the patient’s ability to perform daily activities. The average time from presentation to death is 8.5 years [1, 4]. Regarding the pathogenesis of AD, the Aβ cascade hypothesis believes that the increase of Aβ42/43 in the brain leads to an imbalance of its ratio to Aβ40. The firstly increased deposition of Aβ42/43 induces the senile plaque deposition afterwards and neurodegeneration simultaneously. Accumulation of Aβ42/43 further activates microglia and inflammatory response and leads to mitochondrial damage, energy metabolism disorder, oxidative stress injury, and activation of apoptotic pathways. In addition, the inflammatory response can activate protein kinase, promote the abnormal phosphorylation of tau protein, and make it aggregate to form double-stranded helical filaments and neurofibrillary tangles, resulting in axonal degeneration and neuronal death [1, 5].

Microglia and astrocytes are the main sources of AD inflammatory mediators, which play both pro-inflammatory and anti-inflammatory roles in the brain through the stimulation and release of cytokines in a microenvironment [4]. Microglia, as the largest player in neuroinflammation in the central nervous system (CNS), have an important role in maintaining an optimal brain microenvironment by detecting and clearing debris and responding to virtually all foreign factors in the brain, which are typically described as danger-associated molecular patterns (DAMPs) or pathogen-associated molecular patterns (PAMPs) [6]. Astrocytes are the most abundant glial subtype in the CNS, and similar to microglia, they play a crucial role in the regulation of neuroinflammation [7]. Activated microglia and astrocytes release pro-inflammatory or anti-inflammatory cytokines, leading to inflammatory or anti-inflammatory responses involved in the progression of AD [7]. There is evidence that moderate inflammation in the early stage alleviates Aβ deposition in AD mice, whereas heavy inflammation in the later stage aggravates AD [8].

Toll-like receptors (TLRs) are type I membrane glycoproteins characterized by a cytoplasmic toll/IL-1 receptor domain (TIR) signal domain and an external antigen recognition domain composed of 19–25 tandem Leucine-rich repeat (LRR) motifs [9, 10]. TLR1, 2, 4, 5, 6, and 10 are expressed on the surface of various nerve cells and migrate to the phagocyte after activation. TLR3, 7, 8, and 9 are expressed in almost all intracellular lumens of cell types, mainly in the endosome and endoplasmic reticulum, and their ligand-binding regions are located in the vesicle lumen [11]. The binding of their ligands to TLRs leads to conformational changes and dimerization, thereby recruiting downstream regulatory proteins, such as myeloid differentiation factor 88 (MyD88) and TIR domain-containing adaptor interferon-β (TRIF) [12, 13]. When TLRs are activated by their respective ligands, they cause downstream enzymatic cascades through MyD88 or TRIF, leading to the translocation of the nuclear factor-κB (NF-κB) to the nucleus and the activation of interferon regulatory factors 3/7 (IRF3/7), and then regulating the transcription of inflammatory factors [14]. The activation of TLRs also triggers non-transcriptional responses, such as phagocytosis, autophagy, cell death, and cytokine production [8, 13].

TLR3 is an important member of the TLR family. Unlike other TLRs, it mainly mediates immune inflammation through TRIF. Through its ligand binding, TLR3 recruits the regulatory protein TRIF, leading to the activation of downstream protein kinases and transcription factors, including NK-κB and IRF3, which regulate the expression of type I interferon (IFNs) and the transcription of inflammatory cytokines [15, 16]. This signal transduction pathway is called the TRIF-dependent pathway [10]. Substantial evidence has demonstrated that TLR3 plays an important role in neuroinflammation and neuroprotection in the processes of several neurological diseases, including nervous system tumors [17, 18], neuronal development and brain function [19], viral encephalitis [20], and ischemic/hypoxic brain injury [19, 21, 22]. The activation of TLR3 significantly enhances the phagocytosis of immune cells and tissue repair functions, including the production of a variety of neuroprotective mediators [23].

Research in recent years has found that a sustained immune inflammatory response is one of the core pathological features of AD [24–26]. It suggests that an appropriate amount of inflammatory response in the early stage is conducive to the phagocytosis and clearance of Aβ, but the late and excessive inflammation can aggravate Aβ deposition and neuronal death [27, 28]. Given the effect of TLR3 on the regulation of neuroinflammation and the role of neuroinflammation in AD, we hypothesize that the activation of TLR3 in the early stage of AD reduces its pathological progression and neurological dysfunctions. In the present study, Poly (I:C), a TLR3 ligand, was used to treat APPswe/PSEN1dE9 transgenic mouse starting from the age of 4 to 9 months. Behavioral tests, histological and immunohistochemistry (IHC) staining, and biomolecular methods were used to verify our hypothesis.

## Methods

### Animals

Male APPswe/PSEN1dE9 transgenic (AD, App + Psen1 +) mice and male C57BL/6 J (wild-type, WT) mice were purchased from GemPharmatech Co., Ltd., China, at 8–10 weeks of age. All mice were bred at the animal facility of Xuzhou Medical University and kept in the same housing conditions (temperature, 24 ± 2 °C; humidity, 50% ± 10%; 12 h light–dark cycle; food and water were available ad libitum). The experimental protocol was approved by the ethics committee of Xuzhou Medical University and performed following the guidelines of the Institutional Animal Care and Use Committee of Xuzhou Medical University. The mice aged 4 months were assigned into four groups: (1) WT + Saline (*n* = 15), (2) WT + Poly(I:C) (*n* = 15), (3) AD + Saline (*n* = 15), and (4) AD + Poly(I:C) (*n* = 15).

### Drug administration

Poly(I:C), which was obtained from Tocris Bioscience (Minneapolis, MN, USA), was dissolved in 0.9% NaCl to a concentration of 5 mg/ml and stored at − 20℃. Poly(I:C) (mg/kg body weight) was injected intraperitoneally every 4 days from 4 to 9 months old in the Poly(I:C) groups. The mice in the saline control groups were injected with the same volume of normal saline (0.9% NaCl).

### Western blot analysis

Western blot analysis was performed according to a previous process described [29]. In brief, the mice were decapitated after deep anesthesia, and the brain tissues were harvested from the cortex and the hippocampus and stored at − 80℃. Proteins from the left cortex and hippocampus were extracted with a lysis buffer containing protease and phosphatase inhibitors. The tissues were homogenized and centrifuged (12,000 r/min) for 20 min. The supernatants were collected, and the protein concentrations were detected using a BCA Protein Assay (Beyotime Biotech, Inc.). Proteins were isolated with 8% or 10% sodium dodecyl sulfate–polyacrylamide gel electrophoresis (SDS-PAGE) gels and transferred to a 0.45-µm polyvinylidene difluoride (PVDF) membrane (Millipore IPVH00010, MA, USA) activated with methanol. The membrane was blocked with 5% nonfat dry milk or 5% bovine serum albumin and then incubated with a primary antibody at 4 °C overnight. After being washed with washing buffer, the PVDF membrane was incubated with peroxidase-conjugated secondary antibodies for 1 h at room temperature. Signals were detected with a high-sig ECL Western blotting substrate (Tanon, Shanghai, China) and scanned with a ChemiDoc Imaging System (Bio-Rad Inc., USA). The antibodies used in this study were anti-Aβ (dilution: 1:1000, CN:60,342–1-Ig, Proteintech), anti-CD206 (dilution: 1:1000, CN:18,704–1-AP, Proteintech), anti-CD68 (dilution: 1:1000, CN:28,058–1-AP, Proteintech), anti-CD16/32 (dilution: 1:1000, CN:ab228971, Abcam), anti-Iba-1 (dilution: 1:1000, CN:1363R, Bioss), anti-IRF3 (dilution: 1:1000, CN:2993R, Bioss), anti-p-IRF3 (dilution: 1:1000, CN:9278R, Bioss), anti-NF-κB (dilution: 1:1000, CN:#8242, Cell Signaling Technology), anti-p-NF-κB (dilution: 1:1000, CN:#3033, Cell Signaling Technology), anti-GFAP (dilution: 1:1000, ab7260, Abcam), anti-β-actin (dilution: 1:5000, CN:ab822387, Abcam), and anti-GAPDH (Dilution: 1:5000, CN: 60004–1-Ig, Proteintech).

### IHC staining

IHC staining was performed according to a previously described process [8]. Briefly, the mice were transcardially perfused with 0.9% saline, followed by 4% paraformaldehyde (PFA). The brains were removed and post-fixed in 4% PFA for 24 h at 4 °C and then dehydrated in 15% sucrose for 24 h, followed by 30% sucrose for 48 h. The brains were cut into coronal sections (25-μm thickness) with a rotary frozen slicer (CM1950, Leica, Germany) and stored in a cryoprotectant. The sections were washed three times with 0.3% Triton X-100 in phosphate-buffered saline (PBST). Each section was blocked with 5% goat serum in 0.3% PBST for 2 h at room temperature and then incubated overnight with primary antibodies at 4 °C. After being washed with 0.3% PBST, the sections were incubated with AlexaFlour® 488 secondary antibody or AlexaFlour® 594 secondary antibody for 2 h at room temperature. The primary antibodies used in this study were anti-beta amyloid (dilution: 1:400, CN:60342–1-Ig, Proteintech), anti-NeuN (dilution: 1:500, CN: ab104224, Abcam), anti-GFAP (dilution: 1:400, CN: ab7260, Abcam), anti-CD68 (dilution: 1:500, CN: ab237968, Abcam), and anti-Iba-1 (dilution: 1:1000, CN: ab178846, Abcam). Neuronal density was measured using ImageJ software and quantified as the ratio of the area occupied by neurons to the image area (1024 × 1024 pixels) at the same threshold.

### Detection of mRNA using a quantitative real-time polymerase chain reaction (Q-PCR)

Q-PCR was performed according to a previously described process [30]. Briefly, total RNA was extracted from the cortex and hippocampus tissues using TRIzol reagent (Invitrogen), and the concentration of each sample was assessed using an ultra-micro spectrophotometer (N60, Implen, Germany). The total RNA was reverse-transcribed to produce cDNA using a PrimeScript™ RT Master Mix (Takara, Code No. RR360A) according to the manufacturer’s protocol. Q-PCR was performed using SYBR® Premix Ex Taq TM II (Tli RNaseH Plus) (Takara, Code No. RR820A) on a CFX96™ Real-Time PCR Detection System (Bio-Rad). The amount of RNA was calculated using the 2^− ΔΔCt^ method. The primers were synthesized by Sangon Biotech (Shanghai, China). The sequences of the primers used in the present experiment are listed in Table 1.

**Table 1:** The sequences of primer used in the present experiment

Target genes	Forward	Reverse
IL-1β	5′-GCAACTGTTCCTGAACTCAACT-3′	5′-ATCTTTTGGGGTCCGTCAACT-3′
IL-4	5′-GGTCTCAACCCCCAGCTAGT-3′	5′-GCCGATGATCTCTCTCAAGTGAT-3′
TNF-α	5′-GACGTGGAACTGGCAGAAGAG-3′	5′-TTGGTGGTTTGTGAGTGTGAG-3′
IFNβ	5′-CAGCTCCAAGAAAGGACGAAC-3′	5′-GGCAGTGTAACTCTTCTGCAT-3′
β-actin	5′-GGCTGTATTCCCCTCCATCG-3′	5′-CCAGTTGGTAACAATGCCATGT-3′

### Morris water maze (MWM)

Mice aged 14 months in each group were used for the MWM test to evaluate spatial learning and reference memory according to the processes used in our laboratory [29]. The MWM system (Zhenghua Biological Instruments, China) contains a circular pool with an inner diameter of 122 cm and a water depth of 40 cm. The circular pool was painted white on all sides. A circular platform with a rough white surface (6 cm in diameter) was placed in the middle of the first quadrant (target quadrant) and immersed 1 cm below the surface of the water. Four different black signs were bored at the center of each quadrant wall as spatial reference cues. The MWM test included the place navigation processes (the first to seventh days) and the probe test (the eighth day). During the place navigation period, each mouse was tested in each quadrant once a day (60 s each), with a 20-min break between trials. The mice were placed in the water from each quadrant in random order each day and facing the walls. If a mouse arrived on the platform successfully within 60 s, it was allowed to remain on the platform for 10 s. If the mouse failed to reach the platform within 60 s, it was guided to the platform and permitted to stay there for 10 s. The latency to the platform and the search strategies were recorded in the computer system. After completing the 7-day learning phase, the probe test was conducted on the eighth day, without the platform underwater. The mice were allowed to swim freely for 60 s to search for the location of the original platform from the third quadrant. The time spent in the target quadrant and the number of times they cross over the platform were recorded by system software (Zhenghua Animal Behavior Video Analysis System, China).

### Open field test (OFT)

The OFT was performed as previously described to evaluate the spontaneous locomotor activity and exploratory behavior [8]. The open field system (Zhenghua Biological Instruments, China) consists of four open white stages (50 cm × 50 cm × 50 cm), a video capture, and an analysis system. During the test, each mouse was placed on one side of the stage and allowed to move freely for 5 min. The total traveled distance (mm), the distance traveled in the central area (mm), and the number of excrements was recorded using the video system.

### Elevated plus maze (EPM)

The mice aged 14 months in each group were used for the EPM to assess anxiety and activity according to a previously published method [29]. The EPM system (Zhenghua Biological Instrument, China) was 40 cm above the ground and consisted of two open arms and two enclosed arms that crossed, a video acquisition system, and an analysis system. Inside the maze, the light intensity was set to 45 Lux. During the experiment, the mice were placed at the center of the maze and allowed to explore the maze freely for 5 min each time. The total distance was recorded using a video analysis system (Zhenghua Biological Instruments, China).

### Tail suspension test (TST)

The mice aged 14 months in each group were used for the TST to evaluate depressive behavior according to a previously published method in our laboratory [8]. During the test, the mice were tied to the tip of their tail with a nylon rope under inescapable but moderate pressure. The mice were suspended, one at a time, 40 cm above the ground for 6 min. The video analysis system (Zhenghua Biological Instruments, China) was used to record the rest time during the 6-min period.

### Statistics

All data are presented as the mean ± SEM values. All statistical analyses were performed using GraphPad Prism 8.0. The Shapiro–Wilk test was used to test the normality of the data. The homogeneity of variance of the data was tested by Bartlett’s test. Tukey’s test for post hoc comparisons was used to test the data meeting the homogeneity of variance. Dunnett’s T3 post hoc test was used to test the data with uneven variance. Data on escape latency and edge-type search strategies were analyzed using repeated measures analysis of variance.* P* < 0.05 was considered statistically significant.

## Results

### Administration of AD + Poly(I:C) did not alter survival in WT and AD mice

**Fig. 1:**
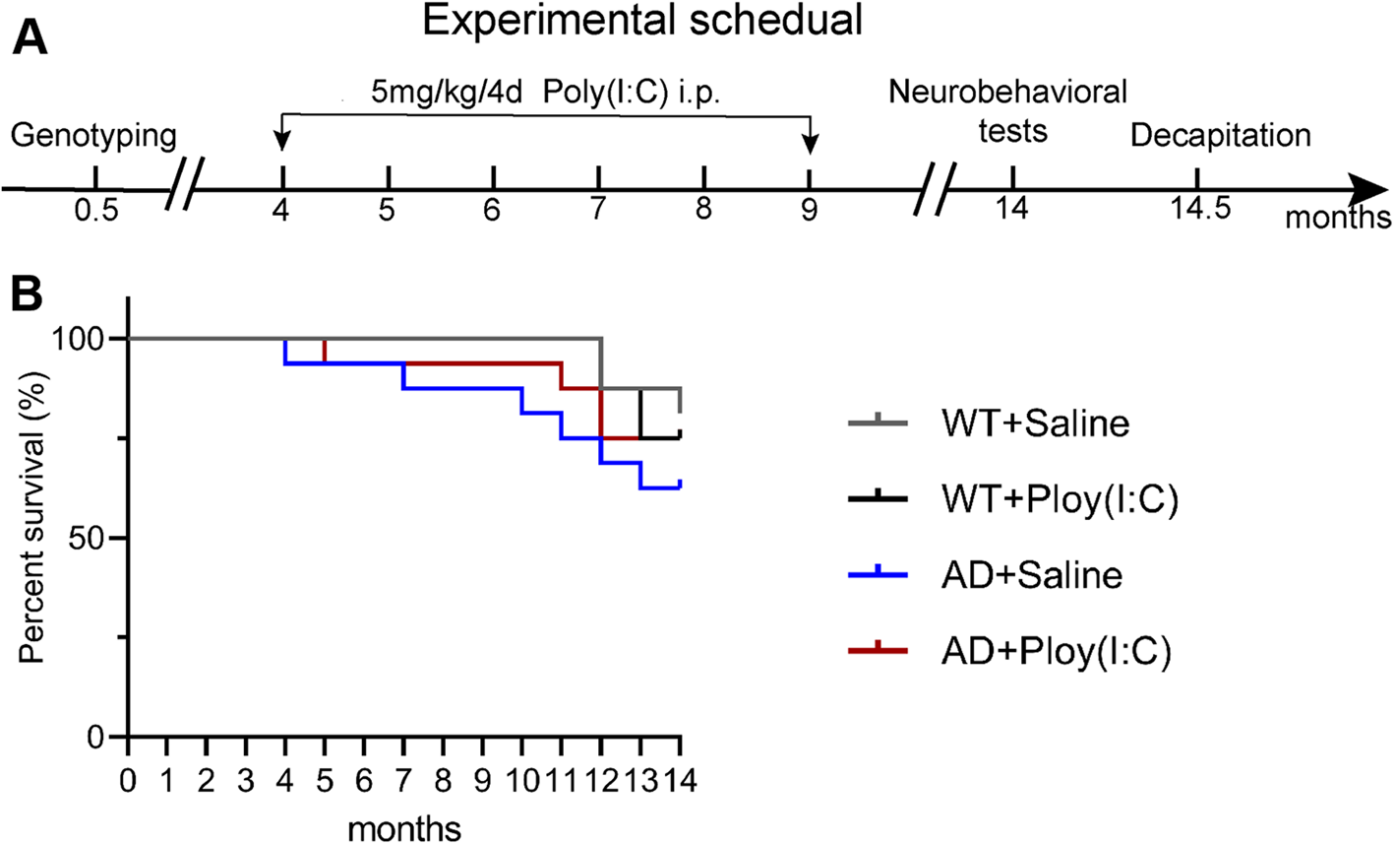
Experimental design and survival rates of the mice in each group. **A** Experimental design: gene identification was performed on the mice. From 4 to 9 months of age, the mice in the treatment groups were administrated with Poly(I:C) through intraperitoneal injection at 5 mg/kg/4 days for 4 months. A battery of behavioral tests was performed, starting at 14 months old. The mice were killed by decapitation under deep anesthesia, and brain tissues were harvested for subsequent experiments at 14.5 months of age. **B** Survival rates of the mice in four groups’ post-treatment period were calculated. No statistical significance was found (*P* > 0.05)

The experimental design plan is shown in Fig. 1A. The mice were injected with drugs starting at 4 months of age and every 4 days for 5 months. The mice were then freely reared to the age of 14 months, and behavioral tests were started. The survival rates of the four groups of mice in each post-treatment period were calculated. No statistical significance was found among them (*P* > 0.05, Fig. 1B).

### Early activation of TLR3 reduced anxiety and depression and increased spontaneous activity in AD mice

**Fig. 2:**
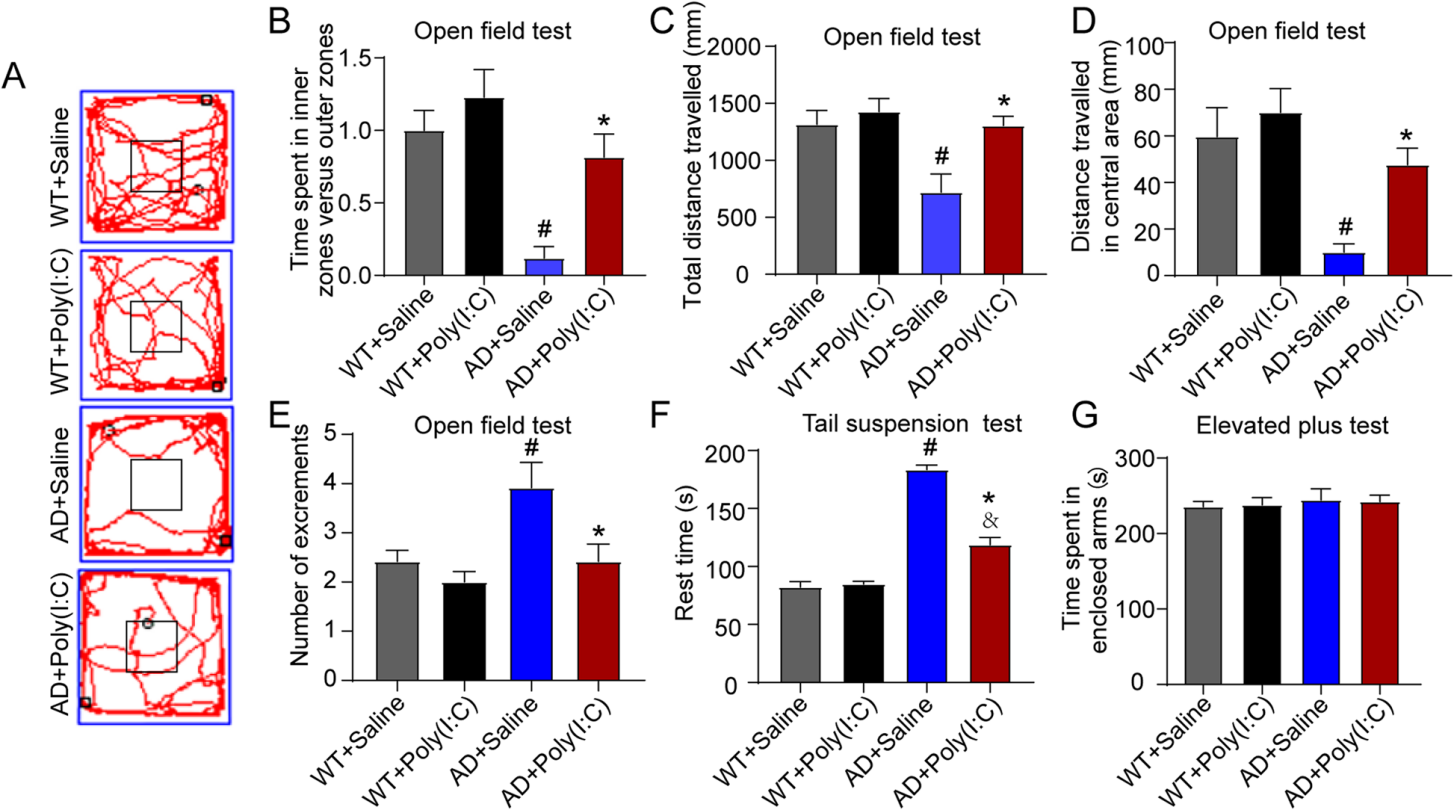
Early activation of TLR3 reduced anxiety and depression and increased spontaneous activity in AD mice. **A** Representative track plots in the OFT. **B** The ratio of the time spent in the inner zone versus the outer zone was reduced in AD mice when compared with WT control mice. Poly (I:C) treatment increased the ratio of the time spent in the inner zone versus the outer zone compared with AD control mice. **C** The total travelled distance of AD + Saline mice was shorter than that of WT + Saline mice. Compared with the AD + Saline mice, the total travelled distance of the AD + Poly(I:C) mice was longer. **D** The distance covered in the central area by AD + Saline mice was shorter than that by WT + Saline mice. Moreover, the distance covered in the central area of AD + Poly(I:C) mice was longer than that of AD + Saline mice. **E** The number of excrements in the AD + Saline mice was greater than that in the WT + Saline mice and less than that in the AD + Poly(I:C) mice. **F** In the TST, the resting immobility time was longer in AD + Saline mice and AD + Poly(I:C) mice than in the WT + Saline mice and longer in AD + Saline mice than in AD + Poly(I:C) mice. **G** In the EPM, the time in the enclosed arms did not show a significant difference among the groups (#: WT + Saline vs. AD + Saline, *P* < 0.05; &: WT + Saline vs. AD + Poly(I:C), *P* < 0.05; *: AD + Saline vs. AD + Poly(I:C), *P* < 0.05; *n* = 8–12/group)

As shown in Fig. 2A, the OFT results indicated that compared with the mice in the WT + Saline group, the mice in the AD + Saline group showed a significantly reduced total distance, central area distance, and increased excrement. However, after activating TLR3, the AD mice tended to explore the central area of the setup, moved less in the marginal area, and had less excrement (*P* < 0.05, Fig. 2C–E). In addition, the ratio of the time spent in the inner zone versus the outer zone was reduced in AD mice when compared with WT control mice (Fig. 2B). Interestingly, Poly (I:C) treatment increased the ratio of the time spent in inner zone versus outer zone compared with AD control mice, indicating the decreased thigmotaxis or anxiety-related behavior after Poly (I:C) administration. In the TST, the resting time of the mice in the AD + Poly(I:C) group was significantly reduced compared with that of the AD mice without the activation of TLR3 (AD + Saline) (*P* < 0.05, Fig. 2F). In the EPM test, no significant difference in the time spent in enclosed arms was observed among the four groups (*P* > 0.05, Fig. 2G).

### Early activation of TLR3 improved memory impairment in AD mice

**Fig. 3:**
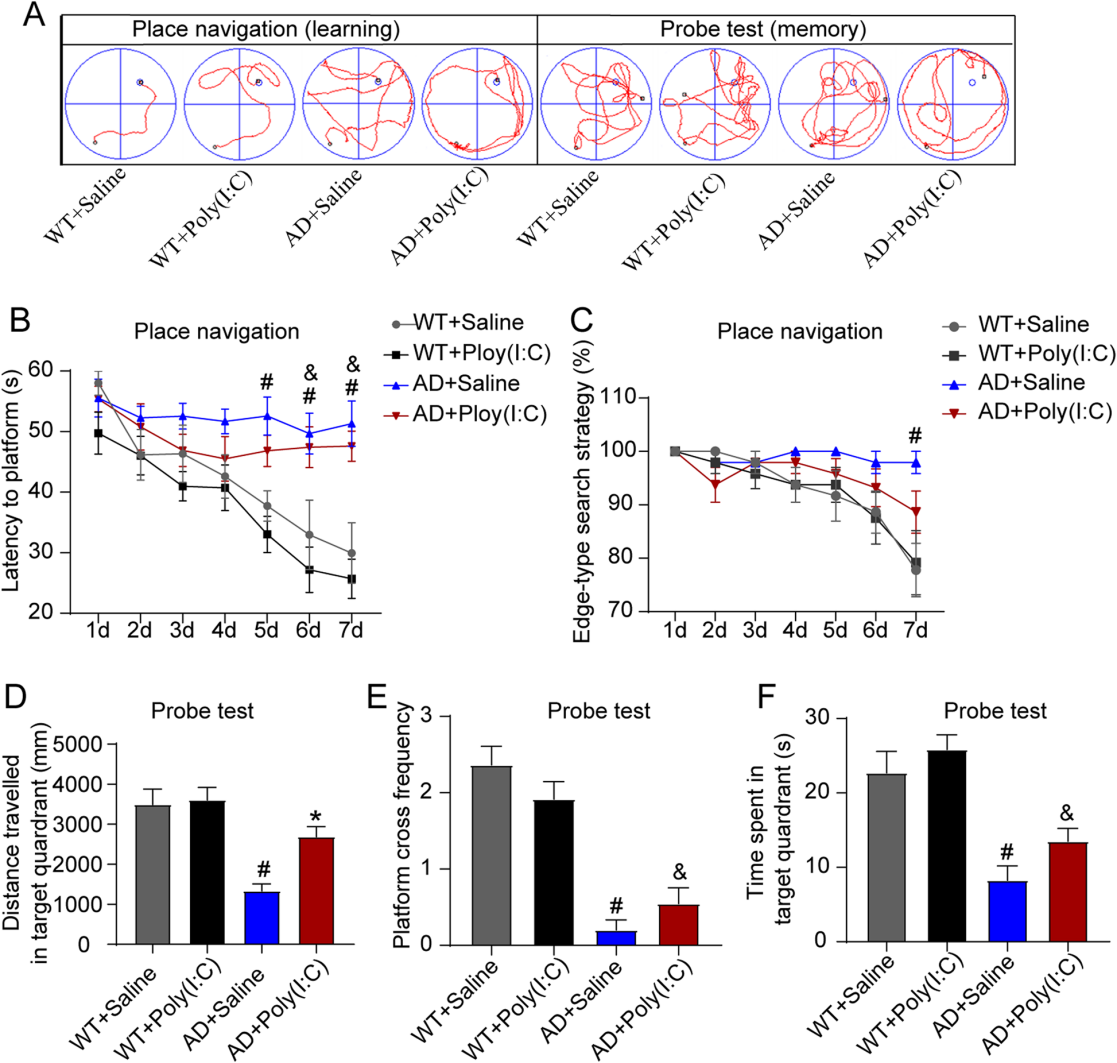
Early activation of TLR3 improved memory impairments in AD mice. **A** Representative track plots in the MWM during place navigation and the probe test. **B** The latency (time to find the hidden platform) increased in the AD + Saline mice and AD + Poly(I:C) mice compared with the WT + Saline mice. **C** As the test progressed, compared with AD + Saline mice, the WT + Saline mice converted the edge-type strategy to the straight-type searching strategy more frequently. **D** In the probe test, the distance covered in the target quadrant was longer in the WT + Saline mice than in AD + Saline mice. However, compared with the AD + Saline mice, the traveled distance in the target quadrant was longer in the AD + Poly(I:C) mice. **E**, **F** The frequencies of crossing the platform area were lower in the AD + Saline mice and AD + Poly(I:C) mice than in the WT + Saline mice. The number of times crossing over the platform area in AD + Saline mice and AD + Poly(I:C) mice was significantly less than that in the WT + Saline mice. No statistical significance was found in the frequencies of crossing the platform area or in the time spent in the target quadrant between AD + Saline mice and AD + Poly(I:C) mice (#: WT + Saline vs. AD + Saline, *P* < 0.05; &: WT + Saline vs. AD + Poly(I:C), *P* < 0.05; *: AD + Saline vs. AD + Poly(I:C), *P* < 0.05; *n* = 8–12/group)

The results from MWM demonstrated that in the acquisition/learning phase, compared with the mice in the WT + Saline group, the AD mice (AD + Saline) took a significantly longer time to find the platform (*P* < 0.05, Fig. 3B). The activation of TLR3 with Poly(I:C) did not significantly change the prolonged time required to find the platform in the AD mice. In the probe test on the eighth day of MWM testing, the mice in the AD + Saline group traveled significantly less distance, spent less time in the target quadrant, and crossed the platform area fewer times compared with the mice in the WT + Saline group (*P* < 0.05, Fig. 3D–F). Interestingly, the distance traveled in the target quadrant in the AD mice treated with Poly(I:C) (AD + Poly(I:C)) was significantly longer than that of the AD mice without Poly(I:C) treatment (*P* < 0.05, Fig. 3D).

### Early activation of TLR3 reduced Aβ deposition in the hippocampus of AD mice

**Fig. 4:**
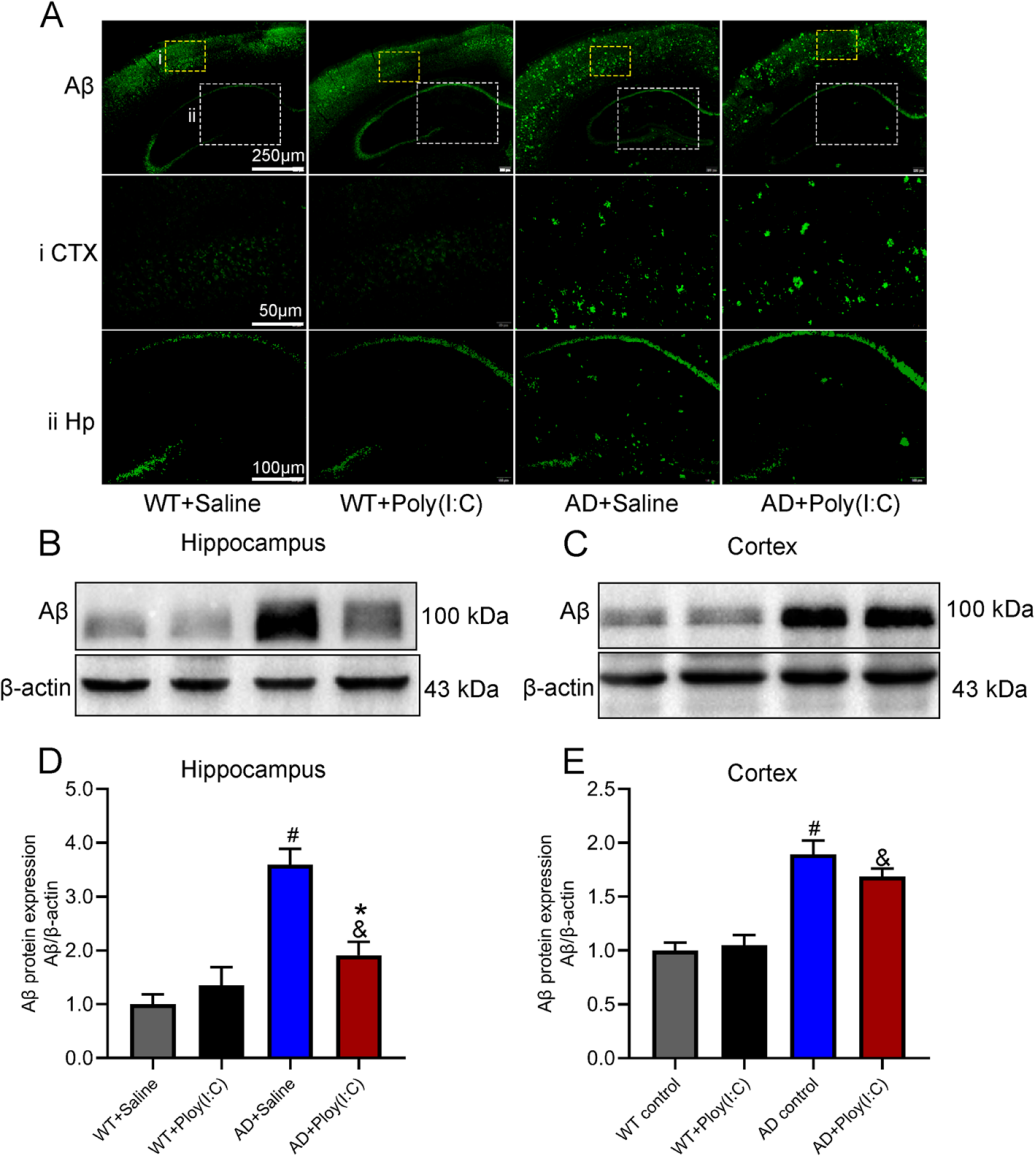
Aβ deposition in mouse brains. **A** Representative immunofluorescence images of Aβ deposition in mouse brains. **B**, **C** Representative Western blot images of Aβ deposition in the hippocampus and cortex. **D**, **E** Results from the quantitative analyses of Western blots showed that the Aβ levels were significantly higher in the AD + Saline mice and AD + Poly(I:C) mice than in the WT + Saline mice. However, compared with the AD + Saline mice, the Aβ levels were significantly lower in the AD + Poly(I:C) mice in the hippocampus but not in the cortex (#: WT + Saline vs. AD + Saline, *P* < 0.05; &: WT + Saline vs. AD + Poly(I:C), *P* < 0.05; *: AD + Saline vs. AD + Poly(I:C), *P* < 0.05; *n* = 4/group)

Aβ deposition is considered one of the pathological features of AD. In the present study, the protein level of Aβ was detected. As shown in Fig. 4, the results from the Western blot and immunofluorescence staining demonstrated abundant Aβ deposition in the cortex and hippocampus of the mice in the AD + Saline and AD + Poly(I:C) groups compared with the WT controls. However, the Aβ level in the hippocampus, not the cortex, was significantly lower in the AD mice treated with Poly(I:C) than in the AD mice without Poly(I:C) treatment (*P* < 0.05, Fig. 4D, E).

### Early activation of TLR3 decreased the activation of microglia in AD mice brains by mediating the polarization of microglia

**Fig. 5:**
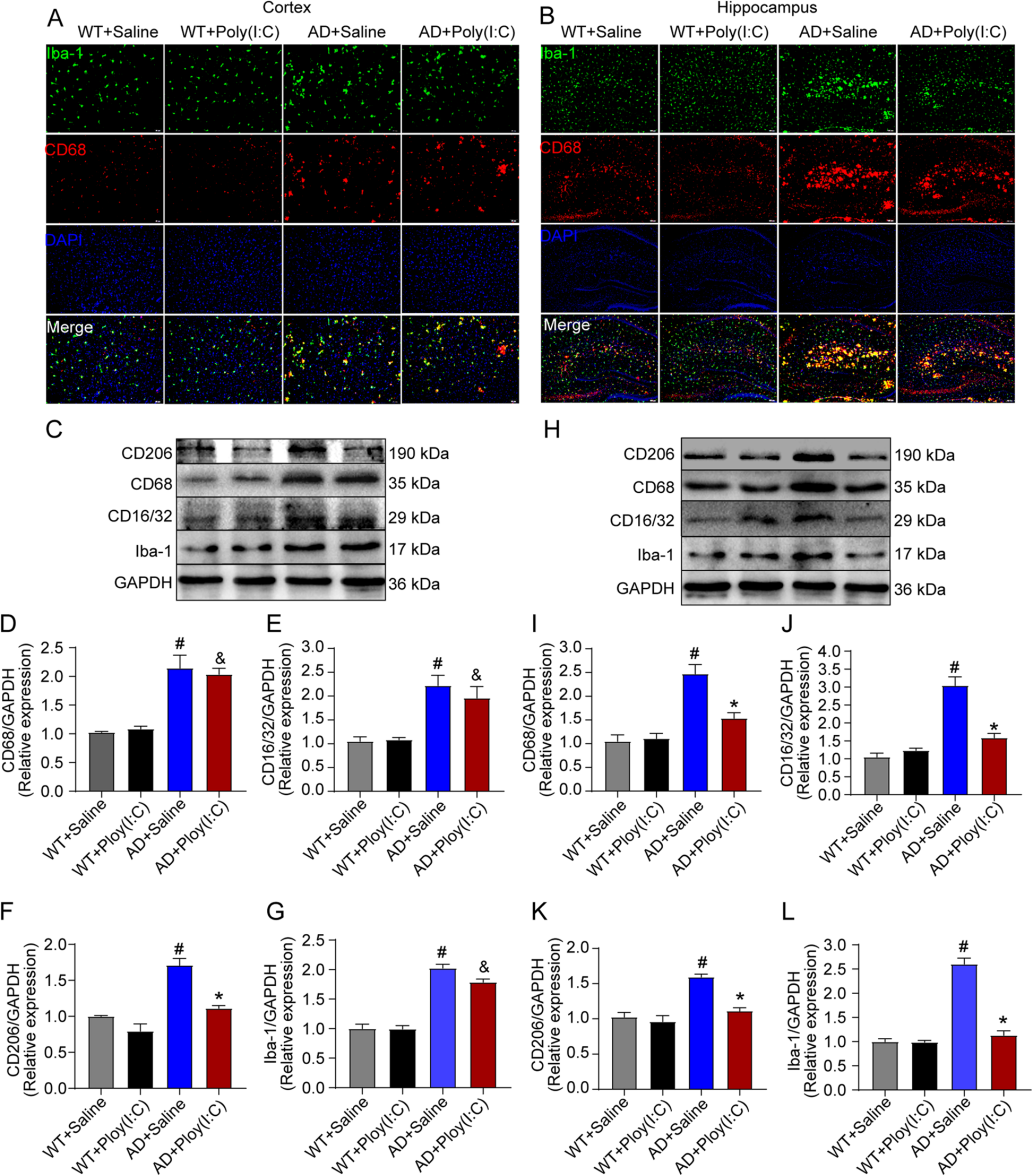
Activation and differentiation of microglia in the hippocampus and cortical areas in mouse brains. **A**, **B** Representative immunofluorescence images of microglial activation for Iba-1 and CD68 staining in the cortex and hippocampus. **C**, **H** Representative images of CD206, CD68, CD16/32, and Iba-1 detected by Western blot in the cortex and hippocampus. **D**–**G** In the cortex, the CD68, CD16/32, CD206, and Iba-1 levels were significantly higher in AD + Saline mice than in WT + Saline mice. The CD68, CD16/32, and Iba-1 levels were significantly higher in the AD + Poly(I:C) mice than in the WT + Saline mice. Compared with those of the AD + Saline mice, the CD206 levels were significantly lower. **I**–**L** In the hippocampus, the CD68, CD16/32, CD206, and Iba-1 levels were significantly higher in the AD + Saline mice than in the WT + Saline mice and were significantly lower in the AD + Poly(I:C) mice than in AD + Saline mice (#: WT + Saline vs. AD + Saline, *P* < 0.05; &: WT + Saline vs. AD + Poly(I:C), *P* < 0.05; *: AD + Saline vs. AD + Poly(I:C), *P* < 0.05; *n* = 4/group)

Chronic inflammation mediated by microglia cells is involved in the pathological process of many chronic degenerative diseases, including Alzheimer’s disease, Parkinson’s disease, and Huntington’s disease [31–33].  In the present study, the protein levels of the markers for the activation of microglia were detected, including ionized calcium binding adaptor molecule 1 (Iba1, a marker of microglia activation), cluster of differentiation 68 (CD68, a prototypical M1 marker), CD16/CD32 (a marker for M1 phenotype microglia), and CD206 (a marker for M2 phenotype microglia). The data showed that the protein levels of Iba1, CD68, CD16/CD32, and CD206 in the hippocampus and cortex of AD mice significantly increased compared with those of the WT controls (*P* < 0.05, Fig. 5). The levels of Iba1, CD68, and CD16/CD32 were significantly reduced in the hippocampus but not in the cortex of AD mice treated with Poly(I:C) compared with AD mice without treatment with Poly(I:C). In addition, compared with untreated AD mice, activating TLR3 with Poly(I:C) significantly reduced the level of CD206 and the transformation from the M1 phenotype to the M2 phenotype in the hippocampus and cortex of AD mice (*P* < 0.05, Fig. 5).

### Early activation of TLR3 reduced the activation of astrocytes in the hippocampus of AD mice

**Fig. 6:**
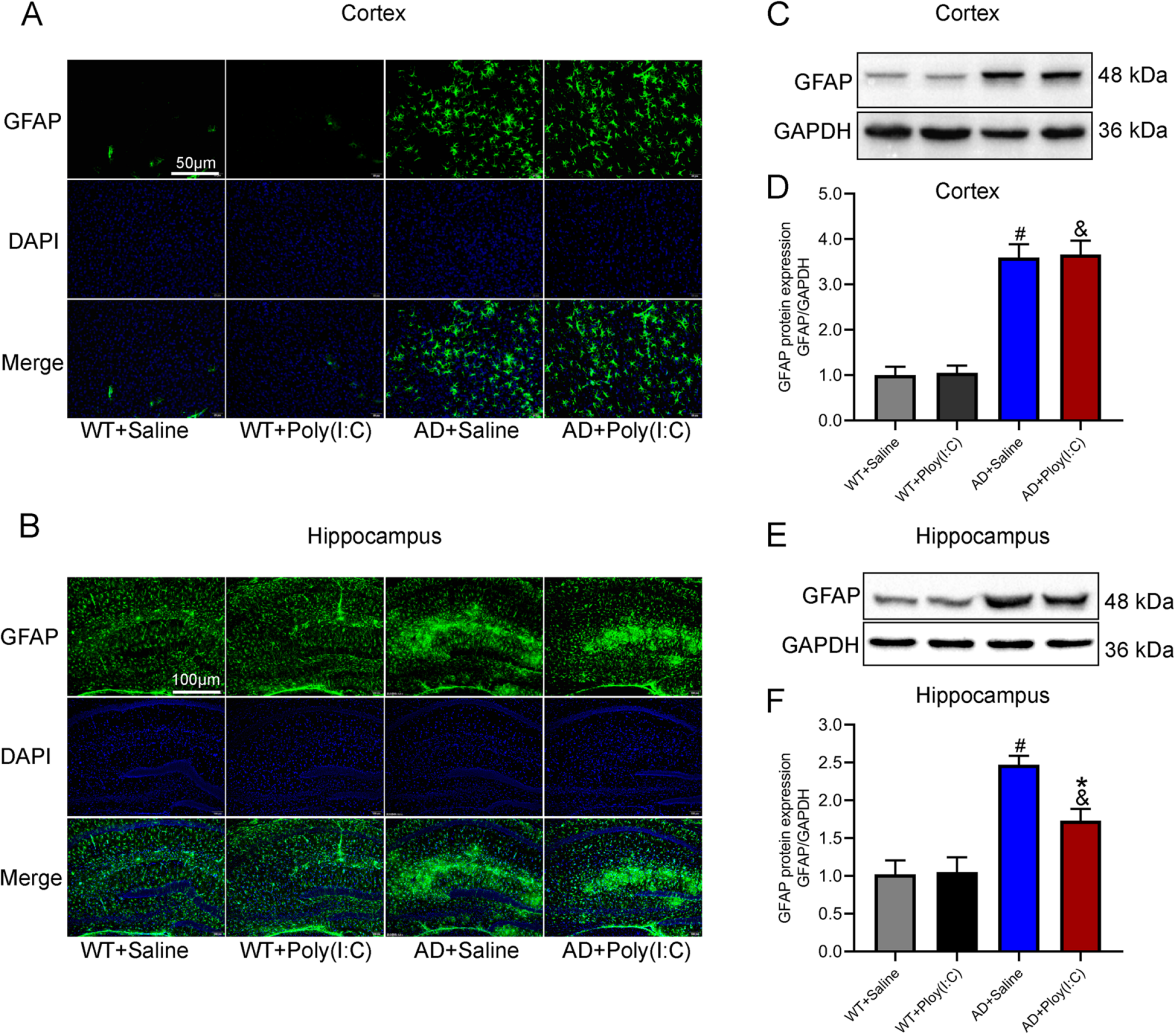
Activation of astrocytes in mouse brains. **A**, **B** Representative immunofluorescence images of GFAP staining in the cortex and hippocampus. **C**, **E** Representative images of GFAP detected by Western blot in the cortex and hippocampus. **D** The GFAP levels were significantly higher in AD + Saline mice and AD + Poly(I:C) mice in the cortex than in the WT + Saline mice. **F** In the hippocampus, compared with the WT + Saline mice, GFAP levels were significantly higher in AD + Saline mice and AD + Poly(I:C) mice and lower in AD + Poly(I:C) mice than in AD + Saline mice (#: WT + Saline vs. AD + Saline, *P* < 0.05; &: WT + Saline vs. AD + Poly(I:C), *P* < 0.05; *: AD + Saline vs. AD + Poly(I:C), *P* < 0.05; *n* = 4/group)

Glial fibrillary acidic protein (GFAP), a specific marker of astrocytes, was detected in this study. As shown in Fig. 6, the mean GFAP levels were elevated in the brains of the mice in the AD + Poly(I:C) and AD + Saline groups compared with the WT controls (*P* < 0.05, Fig. 6). Interestingly, the level of GAFP in the hippocampus but not the cortex of the mice in the AD + Poly(I:C) group was significantly lower than that of the AD + Saline group (*P* < 0.05, Fig. 6).

### Early activation of TLR3 reduced the loss of neurons in AD mice

**Fig. 7:**
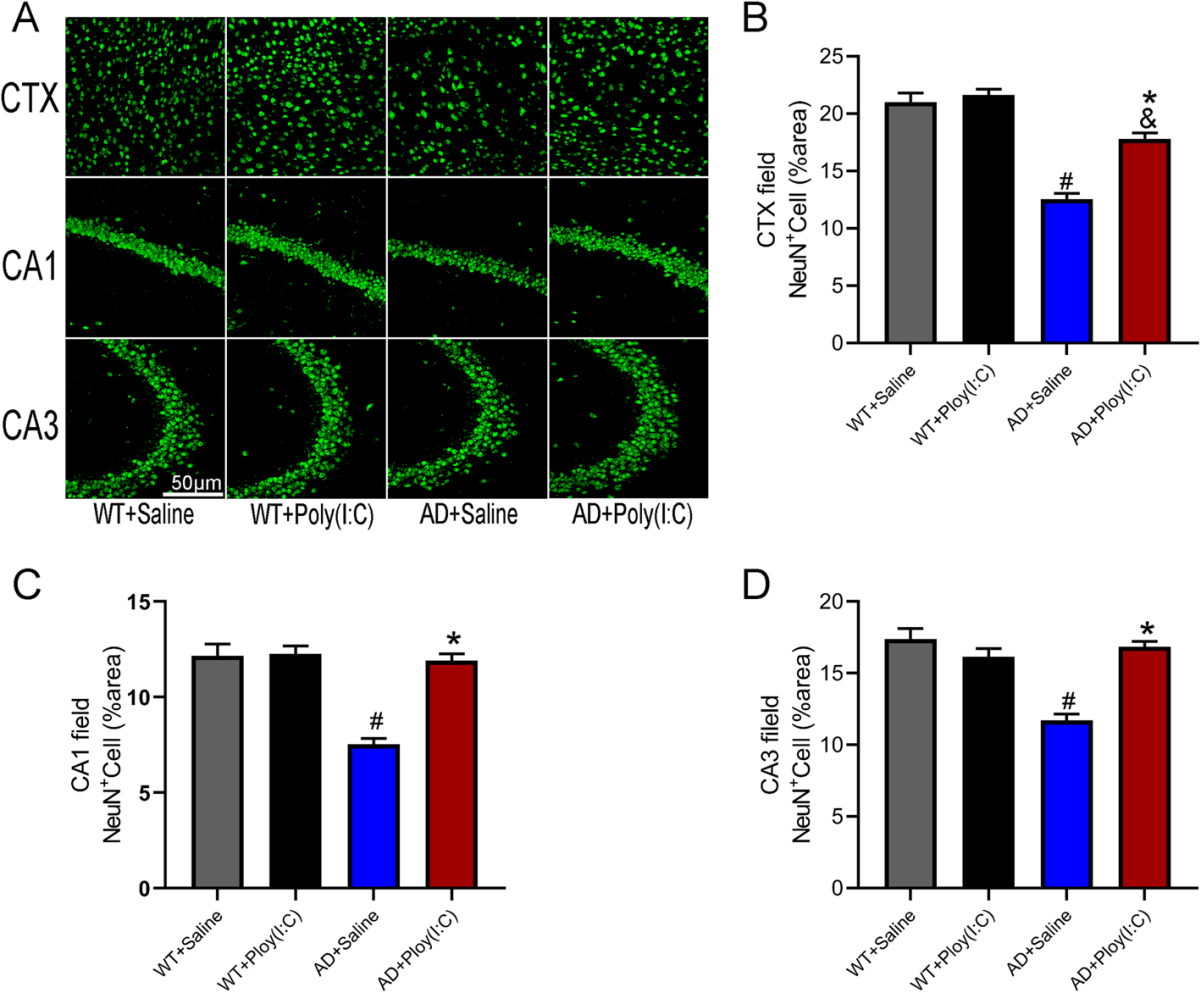
Neuronal loss in mouse brains. **A** Representative immunofluorescence images of neuron staining in CTX, CA1, and CA3. **B**–**D** Neurons in the CTX region were significantly reduced in AD + Saline (*P* < 0.05) and AD + Poly(I:C) mice (*P* < 0.05), and those in the CA1 and CA3 regions significantly decreased in AD + Saline mice (*P* < 0.05) but not in AD + Poly(I:C) mice compared with WT + Saline mice (#: WT + Saline vs. AD + Saline, *P* < 0.05; &: WT + Saline vs. AD + Poly(I:C), *P* < 0.05; *: AD + Saline vs. AD + Poly(I:C), *P* < 0.05; *n* = 4–6/group)

The neuronal number was evaluated using immunofluorescence staining for NeuN (Fig. 7) in the cortical region (CTX) and hippocampus CA1 and CA3. The results showed that the number of neurons in the CTX, CA1, and CA3 of AD mice was significantly reduced compared with that of the WT controls (*P* < 0.05, Fig. 7). However, the number of neurons in the CTX, CA1, and CA3 of AD mice treated with Poly(I:C) was significantly greater than that in AD mice without treatment (*P* < 0.05, Fig. 7).

### Activation of TLR3 inhibited the increased levels of interleukin 1β (IL-1β) and tumor necrosis factor alpha (TNF-α) mRNA and upregulated the decreased interleukin 4 (IL-4) mRNA in the hippocampus of AD mice

**Fig. 8:**
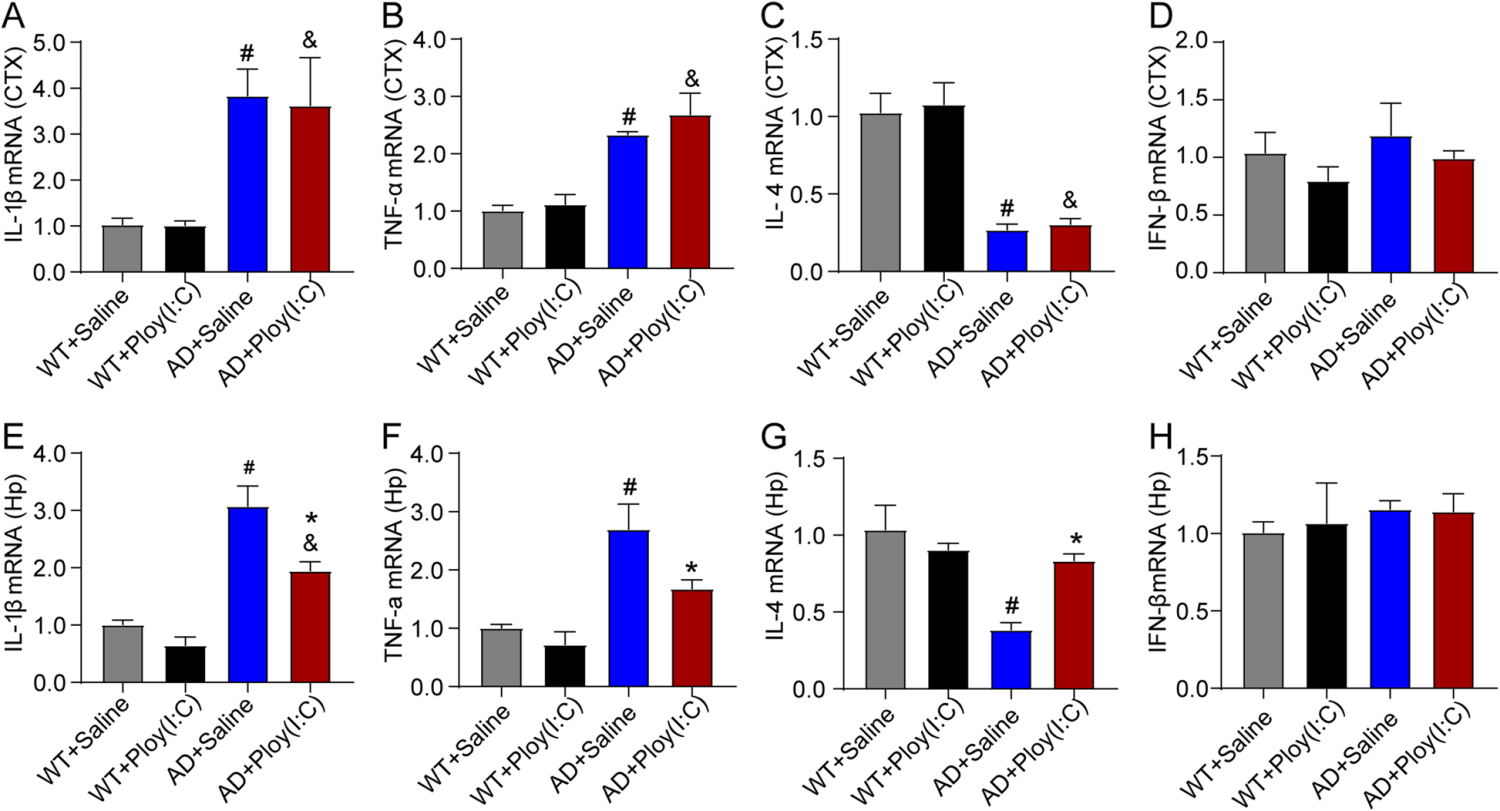
Transcription level of cytokines in mouse brains. **A**, **B** In the cortex, the IL-1β and TNF-α levels in AD + Saline mice and AD + Poly(I:C) mice were significantly increased compared with those in WT + Saline mice (*P* < 0.05). No differences were observed between AD + Saline mice and AD + Poly(I:C) mice (*P* > 0.05). **C** In the cortex, the IL-4 level in AD + Saline mice was dramatically decreased in AD + Saline mice and AD + Poly(I:C) mice compared with that in WT + Saline mice (*P* < 0.05). No statistically significant difference was found between AD + Saline mice and AD + Poly(I:C) mice (*P* > 0.05). **E**, **F** In the hippocampus, the IL-1β and TNF-α levels in AD + Saline mice were significantly increased compared with those in WT + Saline mice (*P* < 0.05) and dramatically decreased in AD + Poly(I:C) mice compared with those in AD + Saline mice (*P* < 0.05). **G** In the hippocampus, the IL-4 level in AD + Saline mice was dramatically decreased in AD + Saline mice compared with that in WT + Saline mice (*P* < 0.05) and was significantly upregulated in AD + Poly(I:C) mice compared with that in AD + Saline mice (*P* < 0.05). **D**, **H** No significant difference was found in the IFN-β levels among the four groups in the cortical and hippocampal regions (*P* > 0.05) (#: WT + Saline vs. AD + Saline, *P* < 0.05; &: WT + Saline vs. AD + Poly(I:C), *P* < 0.05; *: AD + Saline vs. AD + Poly(I:C), *P* < 0.05; *n* = 4/group)

The results demonstrated that in both the hippocampus and cortex of AD mice, the mRNA levels of IL-1β and TNF-α significantly increased, while the IL-4 mRNA level significantly decreased (*P* < 0.05, Fig. 8). In the hippocampus but not in the cortex of AD mice treated with Poly(I:C), the IL-1β and TNF-α mRNA levels were significantly lower, and the IL-4 mRNA level was significantly higher compared with those in AD mice without treatment (*P* < 0.05, Fig. 8). However, no significant difference was found in interferon beta (IFN-β) mRNA among the four groups in the hippocampus and cortex (Fig. 8).

### Activation of TLR3 decreased the activation of NF-κB in the hippocampus of AD mice

**Fig. 9:**
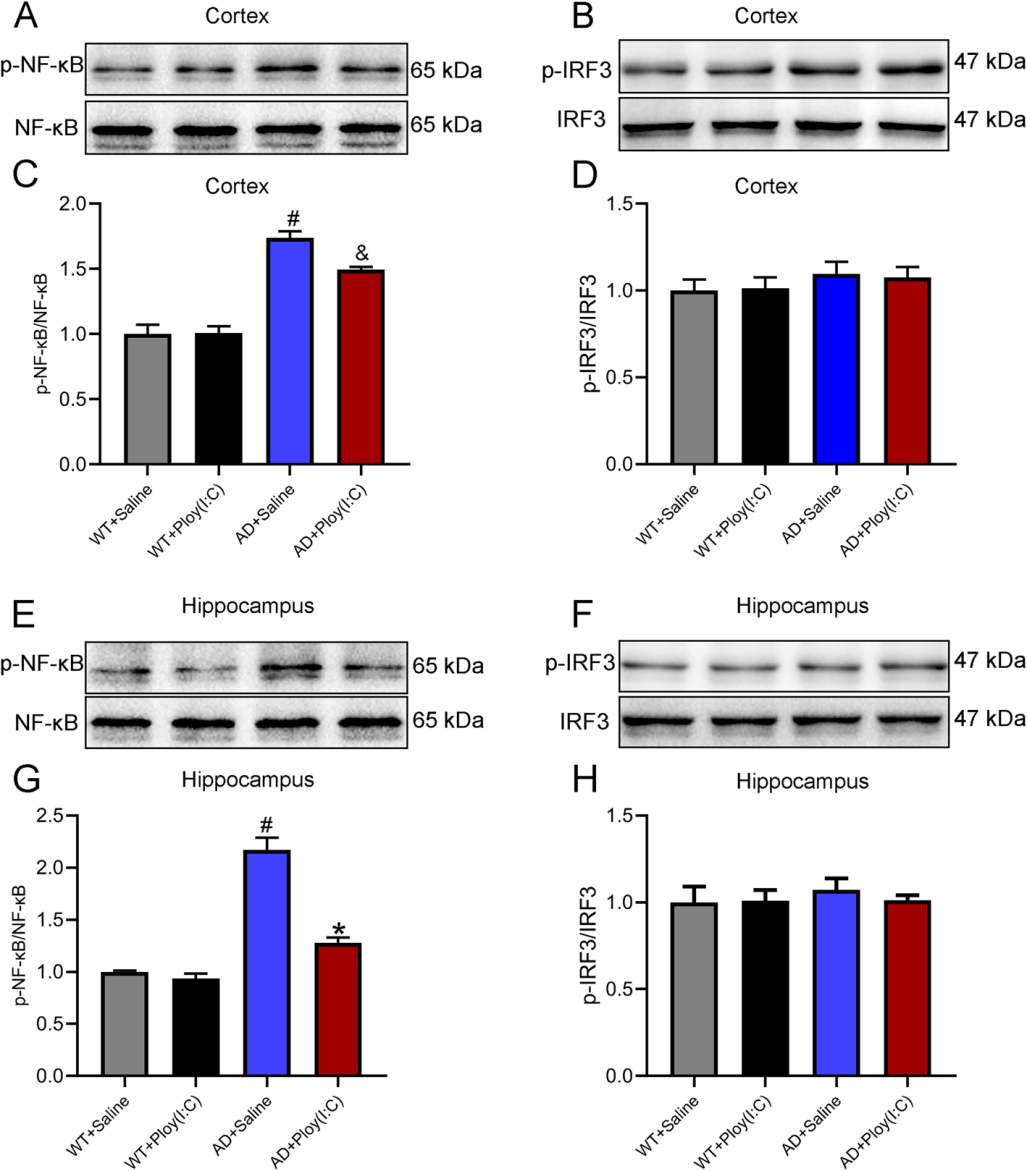
Phosphorylation levels of NF-κB and IRF3 in mouse brains. **A**, **B** Representative Western blot images for NF-κB and IRF3 in the cortex of each mouse. **C** In the cortex, the p-NF-κB level was significantly increased in AD + Saline mice and AD + Poly(I:C) mice compared with that in WT + Saline mice (*P* < 0.05). No significant difference was found between AD + Saline mice and AD + Poly(I:C) mice (*P* > 0.05). **E**, **F** Representative Western blot images for NF-κB and IRF3 in the hippocampus of each mouse. **G** Compared with that of WT + Saline mice, the p-NF-κB level was significantly upregulated in AD + Saline mice (*P* < 0.05) and significantly decreased in AD + Poly(I:C) mice compared with AD + Saline mice. **D**, **H** No significant difference was found in the phosphorylation level of IRF3 among the four groups in the cortical and hippocampal regions (*P* > 0.05) (#: WT + Saline vs. AD + Saline, *P* < 0.05; &: WT + Saline vs. AD + Poly(I:C), *P* < 0.05; *: AD + Saline vs. AD + Poly(I:C), *P* < 0.05; *n* = 4/group)

Activated by the TLR3 ligand, TLR3 activates downstream protein kinases through regulatory proteins, resulting in the phosphorylation of NF-κB and IRF3, regulating the transcription of inflammatory cytokines [10]. In the present study, the phosphorylation of NF-κB and IRF3 in the hippocampus and cortex of mice was detected using Western blot. As shown in Fig. 9, the phosphorylation of NF-κB in the hippocampus and cortex of AD mice was significantly increased compared with that of the WT controls (*P* < 0.05). Moreover, the activation of TLR3 reduced the phosphorylation of NF-κB in the hippocampus (*P* < 0.05) but not in the cortex of AD mice (AD + Poly(I:C)). No significant difference was found in the phosphorylation of IRF3 in the hippocampus and cortex among the four groups (*P* > 0.05, Fig. 9).

## Discussion

Several hypotheses have been made about the etiology and pathological mechanisms of AD, including genetics, β-amyloid cascade, abnormal phosphorylation of tau protein, inflammation, oxidative stress, microcirculation disorders, and cholinergic hypothesis [34–36]. AD is considered to have two characteristic core pathological changes: Aβ deposition and NFTs. The deposition of Aβ in the brain triggers a series of pathological processes and further promotes the deposition of Aβ, forming a cascade amplification reaction. The main component of NFTs is abnormally phosphorylated microtubule-associated tau protein, which is located in the cytoplasm of neurons and causes neurotoxicity [37].

Recent studies have shown that the persistent immune inflammatory response in the brain is the third core pathological change in AD. The continuous activation of microglia and other immune cells in the brain plays an important role in the pathological process of AD [26, 28].The immune inflammatory response is related to the release of activated microglia and a variety of cytokines. The imbalance between anti-inflammatory and pro-inflammatory signals promotes the occurrence and development of AD [24, 25, 38]. The immune inflammatory response is a complex and dynamic process that has different or even opposite effects in different stages of the course of AD. At present, an appropriate amount of inflammatory response in the early stage is considered conducive to the phagocytosis and clearance of Aβ, while a late and excessive inflammatory response can aggravate its deposition and neuronal death [28].

TLRs are a superfamily of type I transmembrane receptors, and they play an important role in the induction and regulation of the immune inflammatory response. Currently, at least 13 TLRs have been found in mammals [39]. TLRs recognize different PAMPs (e.g., lipids, lipoproteins, proteins, and nucleic acids) and DAMPs (endogenous molecules released by immune cells activated by damaged or necrotic tissue), and they activate downstream protein kinases through the regulatory proteins, leading to the activation of NFκB and IRF, thereby inducing and regulating the release of immune inflammatory factors [10, 40]. Therefore, in recent years, the role of TLRs in the inflammatory response and pathological changes in AD has received much attention [41]. However, the experimental results were inconsistent or even contradictory. For example, the genomic deletion of TLR2 induced aggravated white matter damage and deteriorated neurobehavioral functions in mouse models of AD [8]. However, continued TLR2 activation contributes to the developing neuroinflammation and pathology of AD [42]. Pretreatment with low-dose TLR4 agonists induced microglia to produce neuroprotective cytokines and attenuated neuronal degeneration in AD [43]. Double deficiency in TLR2 and TLR4 resulted in subtle impairments in behavioral and cognitive functions [44]. Adipose tissue-derived mesenchymal stem cell–conditioned medium improved memory deficit, decreased Aβ formation, increased neuron survival, and attenuated inflammation by reducing the expression of TLRs [45]. The long-term activation of TLR9 induced a favorable degree of innate immunity stimulation without producing excessive or sustained inflammation, resulting in the efficient amelioration of both cerebral amyloid angiopathy and tau AD-related pathologies in association with behavioral improvements and in the absence of microhemorrhages in aged elderly squirrel monkeys [46]. Nevertheless, these results strongly suggest that TLRs play an important role in AD. However, the role of TLR3 in the neuroinflammation and pathologies of AD has not been investigated.

TLR3 is a member of the TLR family that recognizes double-stranded RNA in endosomes. Unlike other TLRs, TLR3 uses TRIF as the sole adaptor. Upon binding with ligands, TLR3 induces the activation of IRF3 and NF-κB, thereby regulating the inflammatory response. TLR3 has been reported to show a protective role in mouse models of atherosclerosis [47] and contribute to the ischemic preconditioning-induced protection against brain ischemia and attenuation of reactive astrogliosis [48, 49]. The activation of TLR3 24 h before a middle cerebral artery occlusion showed neuroprotective effects against cerebral ischemic injury [50].

In the present study, we applied Poly (I:C), a specific agonist, to activate TLR3 in a male APPswe/PSEN1dE9 transgenic mice model of AD (App + Psen1 + , AD mice) to evaluate the effect of activated TLR3 on neuropathology and neurobehavioral functions in AD and their underlying mechanisms. In view of the existing research showing that early inflammation is beneficial to the phagocytosis and clearance of Aβ, we administered the TLR3 agonist in AD mice starting from 4 months of age and continuing to 9 months of age to investigate the role of the early activation of TLR3 in the pathological process of AD.

It was reported that 5 mg/kg of Poly (I:C) could induce the activation of TLR3 signaling and that its peak could be maintained at 24–48 h after injection [51]. The injection of Poly (I:C) every 4 days could better maintain the drug concentration in mice [51]. However, a higher dose (12 mg/kg) could induce significant hypothermia [52]. For these reasons, in the present study, we selected a dose of 5 mg/kg per body weight for Poly (I:C) intraperitoneal injections every 4 days from 4 to 9 months of age. Our data indicated that the intraperitoneal injection of Poly (I:C) did not alter the survival of the mice (Fig. 1).

The most obvious clinical feature of AD patients is a gradual decline in cognitive function, and a mouse model of AD exhibits significant neurobehavioral dysfunction [8, 29]. In the present study, to evaluate the effects of early-stage treatment with Poly (I:C) on neurobehavioral dysfunctions in AD mice, the neurobehavioral functions were tested at 14 months of age. We found that AD mice showed a significantly reduced total distance and central area distance, as well as increased testing time in TST (Fig. 2). Moreover, the activation of TLR3 reduced anxiety and depression and increased spontaneous activity in the AD mice (Fig. 2). In addition, treatment with Poly (I:C) improved memory deficits in AD mice, as shown by the probe test in MWM, but not learning ability, as indicated by the place navigation processes in MWM (Fig. 3). Our data further demonstrated that the number of neurons in the hippocampus and cortex of AD mice was significantly less than that of WT mice and that the activation of TLR3 by Poly (I:C) attenuated neuron loss in AD mice (Fig. 7).

AD can be divided into sporadic AD and familial AD (FAD). The majority of AD cases are sporadic and are caused by a variety of etiologies, including genetic, environmental, and metabolic factors. Conversely, FAD is caused by mutations in the presenilin genes (PSEN1 and PSEN2) or the Aβ precursor protein (APP) genes [53]. APP gene mutation can cause a large amount of Aβ to be produced and deposited in various parts of the brain. Presenilin gene mutation can cleave Aβ peptide, resulting in the production of more Aβ and accelerating the development of the disease [1]. The APPswe/PSEN1dE9 transgenic mice used in the present study were double transgenic mice expressing a chimeric mouse/human amyloid precursor protein and a mutant human presenilin 1. We found that Aβ deposition increased in the hippocampus and cortex in AD mouse brains. Interestingly, treatment with Poly (I:C) reduced Aβ deposition in the hippocampus but not in the cortex (Fig. 4). The possible reasons for this phenomenon are that the injection of Poly (I:C) may act primarily on the hippocampus [54] and that the hippocampus is more capable of clearing Aβ than the cortical region.

The deposition of Aβ in the brain has been reported to cause oxidative stress and neuroinflammation, and it plays an important role in the occurrence, development, and pathogenesis of AD [4]. Microglia and astrocytes are the main sources of inflammatory mediators in the brain [7]. Microglia are the largest participant in neuroinflammation in the CNS, constantly monitoring the brain and detecting and clearing debris while maintaining an optimal microenvironment [7, 55]. In the presence of Aβ, microglia are activated and form a barrier around the plaque, with the ultimate goal of preventing the further spread of Aβ and clearing it [56, 57]. However, the accumulation of Aβ subsequently recruits more microglia and increases inflammatory responses, leading to an inflammatory cascade [58], which contributes to the pathogenesis of AD. The activation of microglia in the early stage of AD development is more conducive to Aβ clearance [57, 59] and the subsequent recruitment of microglia. Iba1 is a marker of microglia activation, and CD68 is considered a prototypical M1 marker. CD16 is a low-affinity IgG Fc receptor III (FcR III), and CD32 is FcR II. CD16 and CD32 are expressed in B cells, monocytes/macrophages, NK cells, granulocytes, mast cells, and dendritic cells. CD206, also known as mannose receptor C type 1, is a cell-surface protein abundantly present in selected populations of macrophages and dendritic cells. As for macrophages, CD206 is normally expressed in the M2 subtype but not in the M1 subtype and therefore serves as a useful marker to identify the M2 phenotype. In the present study, we detected the markers for microglia, specifically, Mi and M2. Our data demonstrated that both M1 and M2 microglia were significantly increased in the hippocampus and cortex of AD mouse brains. However, treatment with Poly (I:C) reduced the activation of microglia in the hippocampus but not in the cortex of AD mice (Fig. 5).

Astrocytes also play a role in inflammation in AD. Astrocytes interact with neurons and the cerebrovascular system to maintain nutrient and chemical gradients in the brain [4, 55]. In addition, they maintain calcium levels and potassium homeostasis and provide comprehensive neuronal support [60]. In AD brains, astrocytes are affected, leading to astrocyte dysfunction and inflammation [60, 61]. Recent studies have shown that microglia interact with astrocytes. Activated microglia lead to astrocyte activation, forming a feed-forward loop that is detrimental to the surrounding environment. This provides strong evidence that microglia and astrocytes work together to increase neuroinflammation in AD [62]. Our data demonstrated that the activation of TLR3 with Poly (I:C) inhibited the activation of astrocytes in the hippocampus but not in the cortex of AD mouse brains.

The number of Aβ receptors in AD mice is 2.5 times higher than that in normal mice, and the number of pro-inflammatory factors, such as IL-1β and TNF-α, is also 2.5 times higher than that in normal mice, indicating that Aβ may be positively correlated with neuroinflammation [1]. The TLR signaling pathway plays an important role in the activation and regulation of inflammatory responses. The activation of TLR3, through TRIF, results in the activation and IRF3, leading to the transcription of inflammatory cytokines [15, 16]. The activation of TLR3/TRIF signaling also increases the phosphorylation of NK-κB [10]. In the present study, the phosphorylation of NF-κB and IRF3 and the IFN-β mRNA level in the hippocampus and cortex of mice were detected using Western blot and Q-PCR. The data did not show a difference in IRF-3 phosphorylation and IFN-β transcription among the groups (Figs. 8 and 9). Interestingly, the phosphorylation of NF-κB in the hippocampus and cortex of AD mice was significantly increased compared with that of the WT controls. The possible reasons for this phenomenon are as follows: (1) the inflammatory responses in AD brains are mainly regulated by the TLR-NF-κB signaling, not the TLR-TRIF-IRF-3 signaling; (2) the time point of detection was later; and (3) the Poly (I:C)-induced increase in IRF-3 in the early stage returned to normal levels, while NF-κB was persistently activated in AD brains. In addition, we found that the activation of TLR3 by Poly (I:C) inhibited the phosphorylation of NF-κB in the hippocampus (*P* < 0.05) but not in the cortex of AD mice (Fig. 9). We also found that the transcription of pro-inflammatory factors, IL-1β and TNF-α, increased in AD mouse brains, but the level of IL-4 mRNA, an anti-inflammatory factor, decreased. More importantly, treatment with Poly (I:C) reduced the IL-1β and TNF-α mRNA levels and upregulated the IL-4 mRNA level in the hippocampus of AD mice.

## Limitations

There are some limitations when considering our study. First, the present study investigated the effect of early application of TLR3 agonist on AD; however, the dose-dependent relationship of its effect has not been studied, which is worthy of further study. Second, the molecular mechanisms investigated in this experiment are based on TLR3 agonist intervention and later time points following AD development. To study the changes of the TLR3-mediated signaling pathway, as well as the changes of immune inflammatory cells in peripheral blood immediately after Poly (I:C) treatment, will be more helpful to elucidate its mechanism of action.

## Conclusions

In summary, this study showed that the activation of TLR3 by Poly (I:C) in the early stage of development of AD in a mouse model attenuated neuron loss and improved neurobehavioral functions. The underlying mechanisms may be attributed to Aβ clearance, the inhibition of glial cells, and the regulation of neuroinflammation in the hippocampus. However, the differential effects of Poly(I:C) on the hippocampus and cortex require further investigation.

## Data Availability

The datasets used and/or analyzed during the current study are available from the corresponding author on reasonable request.
